# Impact of fetal exposure to mycotoxins on longissimus muscle fiber hypertrophy and miRNA profile

**DOI:** 10.1186/s12864-022-08794-0

**Published:** 2022-08-16

**Authors:** M. A. Greene, A. N. S. Udoka, R. R. Powell, R. E. Noorai, T. Bruce, S. K. Duckett

**Affiliations:** 1 Department of Animal and Veterinary Sciences, Clemson, USA; 2 Clemson Light Imaging Facility, Clemson, USA; 3 Genomics and Bioinformatics Facility, Clemson University, Clemson, USA; 4 Department of Bioengineering, Clemson University, Clemson, SC 29634 USA

**Keywords:** Sheep, Mycotoxin, Muscle fiber, Hypertrophy, miRNA

## Abstract

**Background:**

Longissimus muscle samples were collected from lambs exposed in utero to mycotoxins [E-, endophyte-free tall fescue seed without ergot alkaloids (negative control) or E + , endophyte-infected tall fescue seed containing ergot alkaloids] during mid-gestation (MID; E + /E-) or late-gestation (LATE; E-/E +) harvested at two developmental stages (FETAL, gestational d133) or (MAT, near maturity, 250 d of age; *n* = 3/treatment/developmental stage). Muscle samples were examined to determine the impact of in utero mycotoxin exposure on skeletal muscle fiber hypertrophy and the miRNA profile at FETAL and MAT.

**Results:**

Longissimus weight was greater (*P* < 0.05) in E + /E- lambs compared to E-/E + lambs at MAT; however, FETAL longissimus weight did not differ (*P* > 0.10) between fescue treatments. Type I fiber cross sectional area was larger (*P* < 0.10) for E + /E- than E-/E + at MAT but did not differ (*P* > 0.10) between fescue treatments at FETAL. Type II fiber area was larger (*P* < 0.05) at MAT in E + /E- compared to E-/E + but did not differ (*P* < 0.05) between fescue treatments at FETAL. Cross-sectional Type I and Type II longissimus muscle fiber area increased (*P* < 0.05) from FETAL to MAT by 6.86-fold and 10.83-fold, respectively. The ratio of Type II:Type I muscle fibers was lower (*P* = 0.04) at MAT compared to FETAL. There were 120 miRNA differentially expressed (q < 0.05) between FETAL and MAT. Maternal fescue treatment did not alter (q > 0.05) expression of miRNAs in the longissimus muscle. miR-133, -29a, -22-3p, and -410-3p were identified as highly significant with a log_2_ fold change > 4. In vitro satellite cell cultures showed that selected miRNAs (miR-22-3p, 29a, 27a, and 133a) are differentially regulated during proliferation and differentiation indicating a role of miRNA in muscle hypertrophy.

**Conclusions:**

Exposure to mycotoxins did not alter fiber type but had long-term impacts on postnatal muscle hypertrophy and cross-sectional area. The miRNA profile of the longissimus was not altered by Maternal mycotoxin exposure at FETAL or MAT. Developmental age altered the miRNA transcriptome and mRNA expression of known genes related to muscle growth. These results indicate that Maternal exposure to E + fescue seed during LATE gestation can alter postnatal muscle hypertrophy in sheep; however, these changes are not regulated by the miRNA transcriptome of the longissimus muscle.

**Supplementary Information:**

The online version contains supplementary material available at 10.1186/s12864-022-08794-0.

## Background

Mammalian myogenesis begins in utero and muscle fiber number is established prior to birth [1, 2]. Ovine muscle development begins with primary myogenesis which is complete day 38 of gestation [3] and is followed by secondary myogenesis which is complete by day 85 – 90 of gestation [4, 5]. After d 85–90, skeletal muscle growth is regulated by hypertrophy of existing muscle fibers. Satellite cells are responsible for hypertrophic growth (increased cross-sectional area) and provide increased DNA content for multinucleated muscle fibers to support greater protein synthesis [6]. Satellite cells are found in the basement membrane of muscle tissue adjacent to the myofibers and are in a state of dormancy [7]. Satellite cell activation is regulated by PAX7 and MSTN transcription factors [8]. These cells when activated will proliferate and differentiate to fuse with existing myofibers for growth and repair of tissue [9].

Secondary myogenesis in hindlimb muscle is influenced by maternal undernutrition [10–12] and offspring that are small for gestational age (i.e. runts) have a lower number of secondary fibers [13]. Muscle hypertrophy in late gestation and during postnatal growth are dependent on satellite cell proliferation and fusion. microRNAs (miRNAs) may be involved in regulating satellite cell activation [14] and proliferation/differentiation [15]. miRNAs are small noncoding RNAs that regulate 60% of protein coding gene expression post transcriptionally [16]. miRNA usually repress gene expression through complementary binding with target mRNAs 3’ untranslated regions [17]. Our previous research has shown that maternal consumption of ergot alkaloids, a class of mycotoxins from endophyte-infected tall fescue [E + ; *Lolium arundinaceum* (Schreb.) Darbysh, infected with *Epichloe coenophiala*], during gestation can alter muscle mass, fiber number, and miRNA expression in the fetal semitendinosus muscle [18]. Additional research found that mycotoxin exposure during late gestation also caused long-term effects on longissimus muscle during postnatal growth [19]. Therefore, the objective of this experiment was to examine the impact of mycotoxin exposure during mid- or late-gestation on longissimus muscle fiber hypertrophy and miRNA transcriptome at two developmental stages (FETAL, gestational d 133 or MAT, near maturity) to better understand how miRNAs are involved in muscle hypertrophy.

## Results

### Lamb and muscle mass

**Fig. 1:**
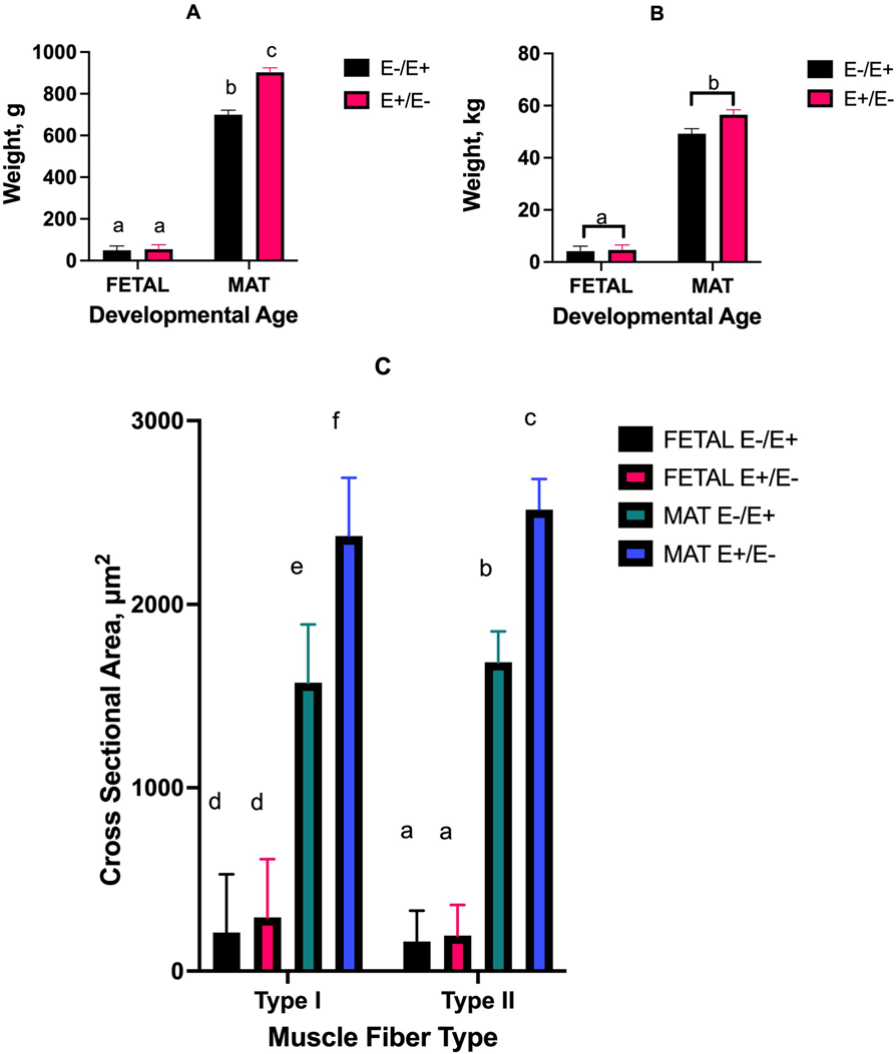
Changes in longissimus muscle weight (**A**), body weight (**B**), and cross-sectional fiber area (**C**) in lambs exposed to mycotoxins (E + /E- vs. E-/E +) at two developmental stages (FETAL, gestational d 133 vs. MAT, near maturity). ^abc^Means with differing superscripts differ *P* < 0.05. ^def^Means with differing superscripts differ *P* < 0.10

The interaction between maternal fescue treatment and developmental stage was significant (*P* < 0.001) for longissimus muscle weight (Fig. 1A). At fetal stage (gestational d 133 or gd133), longissimus muscle mass did not differ by mycotoxin treatment; however, E + /E- (MID) lambs had greater longissimus muscle mass than E-/E + (LATE) lambs at MAT stage. Longissimus muscle mass increased by 1437% from d 133 of gestation to maturity (MAT). Lamb body weight increased (*P* < 0.0001) by 1108% from FETAL (gd133) to MAT (59 kg; Fig. 1B).

### Muscle fiber type

**Fig. 2:**
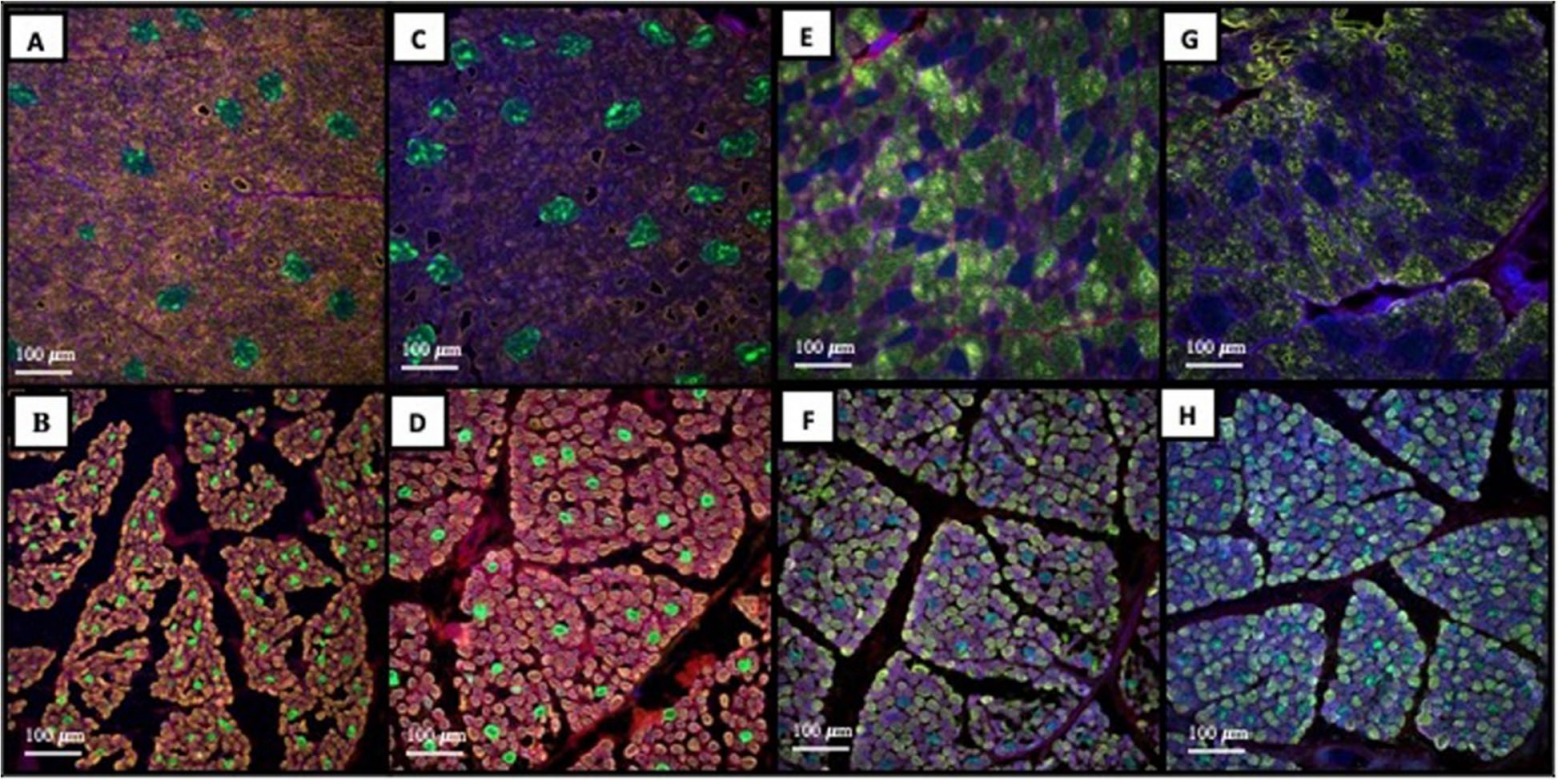
Histology images of the longissimus muscle stained using immunofluorescence for Type I (green) and Type II (orange) muscle fibers by mycotoxin exposure and developmental stage (**A**: MAT, E-/E + ; **B**: FETAL, E-/E + ; **C**: MAT, E + /E-; **D**: FETAL, E + /E-). Histology images of the longissimus muscle stained using immunofluorescence for Type IIa (orange) and IIx (green) muscle fibers (**E**: MAT, E-/E + ; **F**: FETAL, E-/E + ; **G**: MAT, E + /E-; **H**: FETAL, E + /E-)

Muscle fiber histology images are displayed in Fig. 2. The interaction between fescue seed treatment and animal age was significant (*P* = 0.003) for Type II cross sectional area and tended to be significant (*P* = 0.081) for Type I (Fig. 1C). Type I fiber cross sectional area was larger (*P* < 0.10) for E + /E- than E-/E + at MAT but did not differ (*P* > 0.10) by mycotoxin exposure at FETAL age. Type II fiber area was larger (*P* < 0.05) at MAT in E + /E- compared to E-/E + but did not differ (*P* < 0.05) between fescue treatments at FETAL age. Cross-sectional Type I and Type II longissimus muscle fiber area increased (*P* < 0.05) from FETAL to MAT by 6.86-fold and 10.83-fold, respectively. Animal age altered (*P* < 0.05) longissimus muscle fiber types; however, mycotoxin exposure and the interaction with animal age was non-significant (*P* > 0.28) for muscle fiber types (Table 1). MAT lambs had increased (*P* < 0.05) Type I fibers and reduced (*P* < 0.05) Type II fibers as a percentage of the total fibers when compared to FETAL lambs. Within the Type II fiber family, Type IIa and IIx were also examined. MAT lambs had reduced (*P* < 0.05) Type IIa fibers and increased (*P* < 0.05) Type IIx fibers as a percent of the total Type II fibers when compared to FETAL lambs. The ratio of Type II/Type I fibers was reduced (*P* < 0.05) for MAT lambs when compared to FETAL lambs.

**Table 1:** Changes in muscle fiber type and cross-section area in longissimus muscle (LM) of lambs exposed to mycotoxins (E + or E-) during mid- (E + /E-) or late-gestation (E-/E +) and examined at two developmental time points (FETAL or MAT)

**Developmental stage**	**FETAL**		**MAT**		
**In utero mycotoxin exposure (mid/late)**	**E-/E + **	**E + /E-**	**E-/E + **	**E + /E-**	**SEM**
***Fiber type*** Type I, %	8.09^a^	7.11^a^	14.84^b^	10.81^b^	2.244
Type II, %	91.91^a^	92.89^a^	85.16^b^	89.19^b^	2.244
IIa, %	44.49^a^	46.37^a^	32.69^b^	37.07^b^	4.488
IIx, %	55.51^a^	53.63^a^	67.31^b^	62.93^b^	4.488
Ratio II/I	14.02^a^	15.94^a^	7.37^b^	8.79^b^	2.918

^ab^Means in the same row with uncommon superscripts differ by AGE (*P* < 0.05)

### miRNA sequencing

**Fig. 3:**
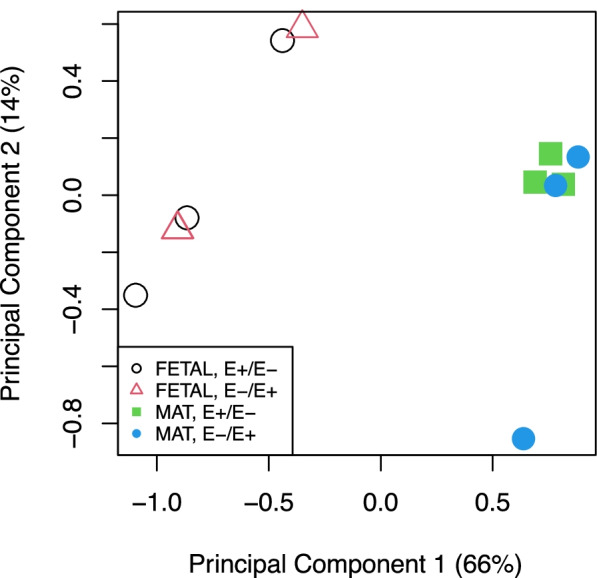
Principle component analysis of miRNA sequencing samples

**Fig. 4:**
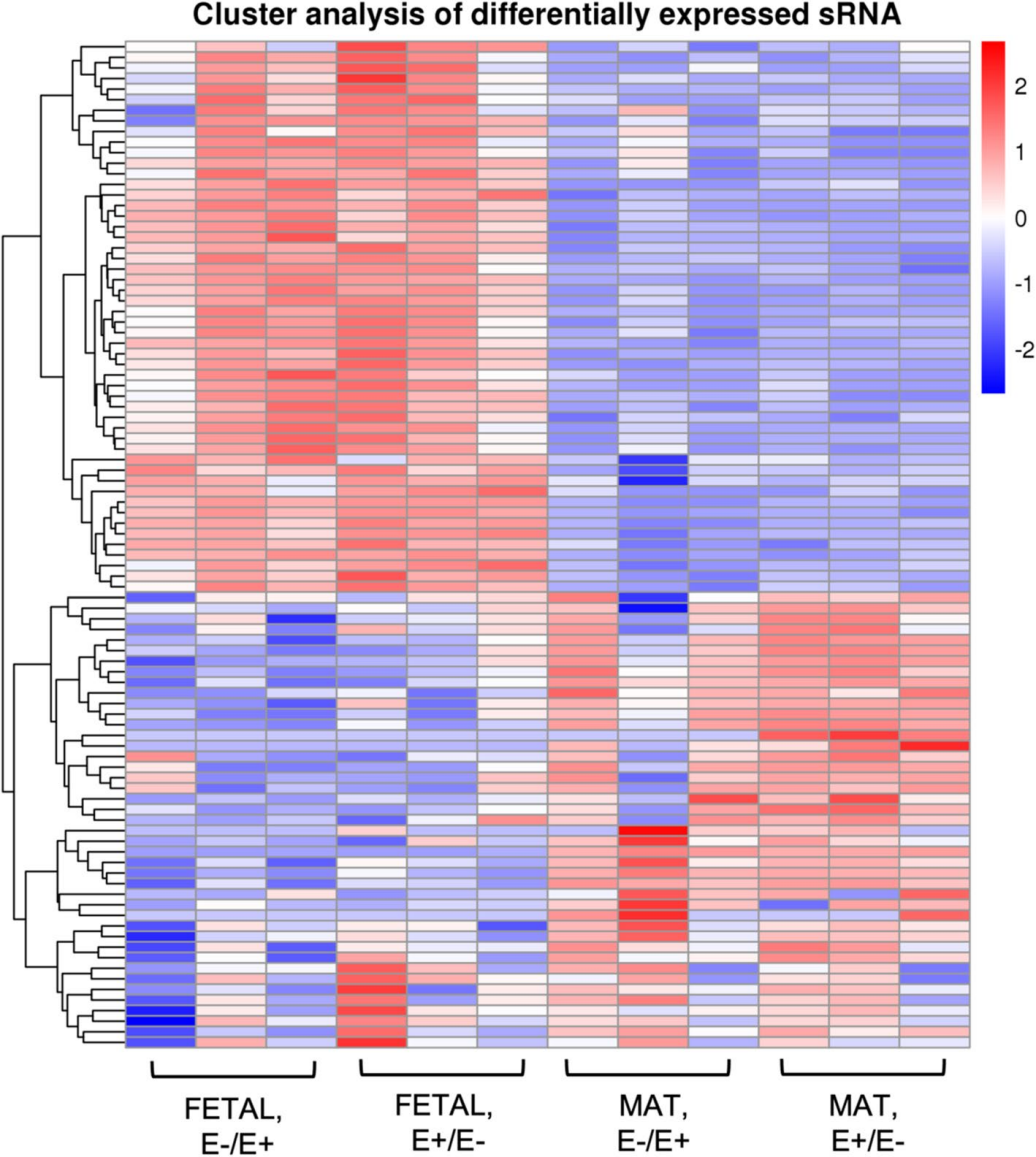
Cluster analysis of differentially expressed miRNA showing the effect of mycotoxin exposure (E + /E- vs E-/E +) and developmental stage (FETAL, gestational d 133 vs MAT, near maturity). Red indicates increased log fold change while blue shows a decreased log fold change

In utero mycotoxin exposure did not alter (q > 0.05) the expression of miRNAs in the longissimus muscle; whereas animal age had a major impact on miRNA expression. Figure 3 displays the principal component analysis showing that FETAL samples and MAT grouped together and maternal mycotoxin treatment does not impact sample clustering. There were 120 miRNAs that were differentially expressed (q < 0.05) between FETAL and MAT age (Fig. 4, Supplemental Table 1). Sixty-three miRNA were upregulated in MAT tissue and 57 were down-regulated in MAT tissue when compared to FETAL tissue. Twenty-one novel miRNAs were identified. One novel miRNA was down-regulated in MAT muscle and 20 were up-regulated in MAT muscle when compared to FETAL.

Current miRNAs sequencing showed a high abundance of miR-133 in all samples, and MAT samples had a 4.02 log_2_ fold change increase (q = 2.44E-13) in expression compared to FETAL samples. The miR-29a expression for MAT samples was 5.22 log_2_ fold change greater (q = 3.93E-27) when compared to FETAL samples. miR-22-3p expression was increased (q = 5.15E-37) by 4.94 log_2_ fold change for MAT samples compared to FETAL samples. Expression of miR-410-3p was reduced (q = 2.66E-23) by 5.19 log_2_ fold change for MAT samples compared to FETAL samples. miR-299-5p was reduced (q = 1.25E-22) by -4.39 log_2_ fold change. The most abundant miRNA at FETAL included miR-148a, -381-3p, and -127 and let-7i and -7f. The most abundant miRNAs at market weight (MAT) were miR-133, -148a, -26a, and -10b and let-7f.

**Fig. 5:**
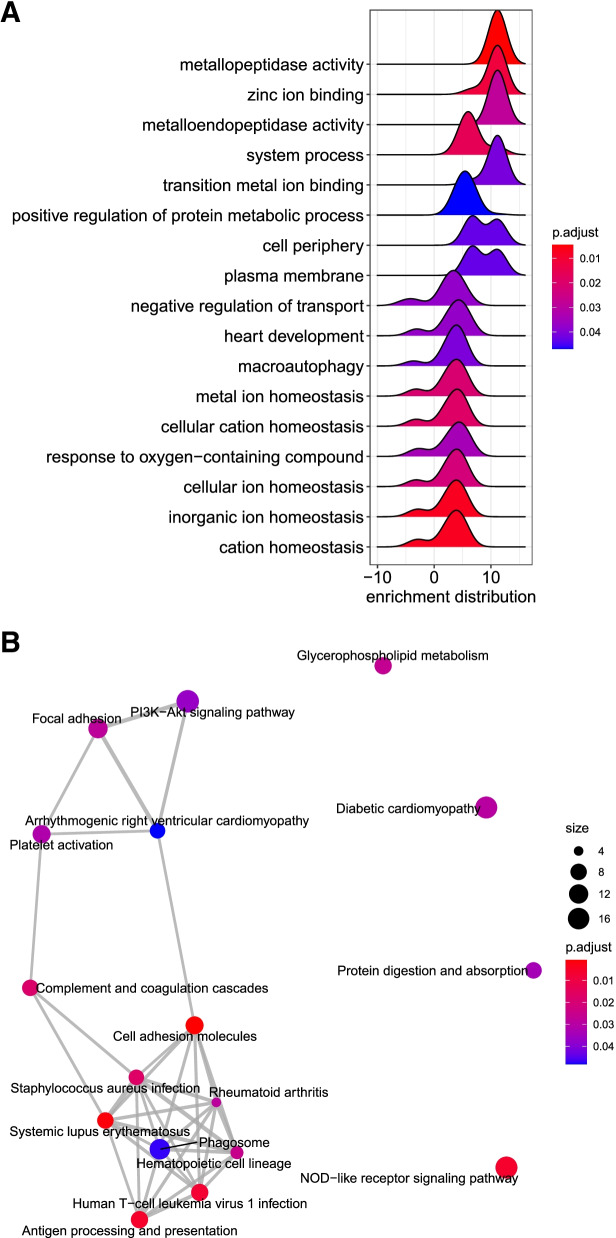
GOseq results of FETAL vs MAT miRNA sequencing predicted mRNA targets of differentially expressed miRNA (**A**). Peak size and location on the x-axis indicate the expression distributions of core enriched genes. *P*_*adj*_ is indicated by the color scale. KEGG pathway analysis clustering of FETAL vs MAT miRNA sequencing predicted mRNA targets of differentially expressed miRNA (**B**). Number of enriched genes in a pathway is indicated by the size of the circles and *P*_*adj*_ is shown by the color scale

**Fig. 6:**
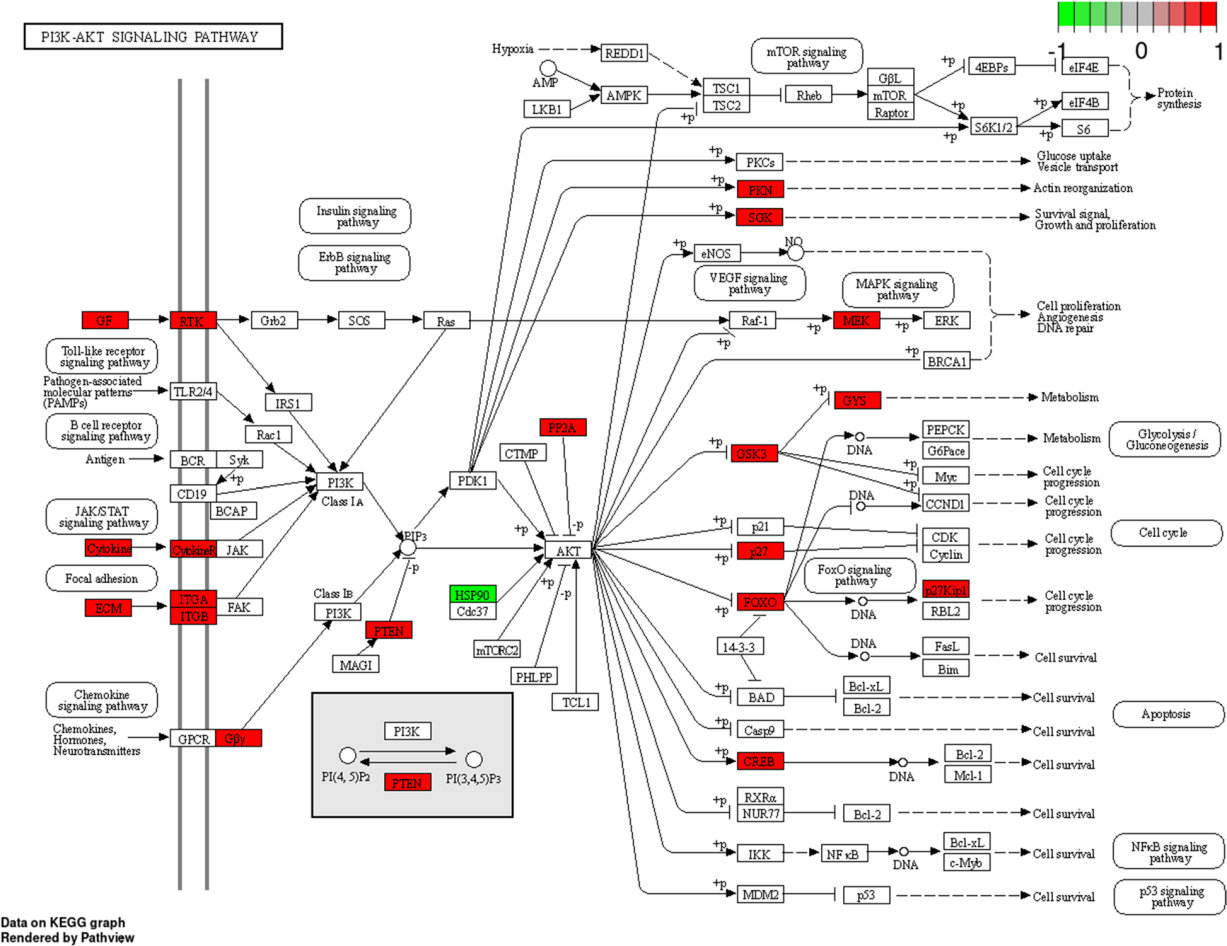
PI3K – ATK Signaling Pathway with enriched genes from KEGG pathway analysis (oas04151; 62). The Kanehisa Laboratories have happily provided permission. Genes positively enriched are colored red and genes negatively enriched are colored in green

A list of target mRNAs was generated from the FETAL vs MAT differentially expressed miRNAs for gene ontology analysis. Seventeen GO-terms belonging to the 3 ontology classes (molecular function, cellular component, and biological process) were enriched (*P* < 0.05) and are displayed in Fig. 5A. The gene targets of the differentially expressed miRNA of FETAL vs MAT were also annotated using the Kyoto Encyclopedia of Genes and Genomes to examine miRNA relationship to cellular pathways. Seventeen different pathways were enriched (*P* < 0.05; Fig. 5B). The Phosphoinositide 3 kinase – Protein Kinase B (PI3K – AKT) Signaling Pathway is a known pathway that is involved in skeletal muscle growth and development and enriched genes are displayed in Fig. 6 [20].

### Gene expression

**Fig. 7:**
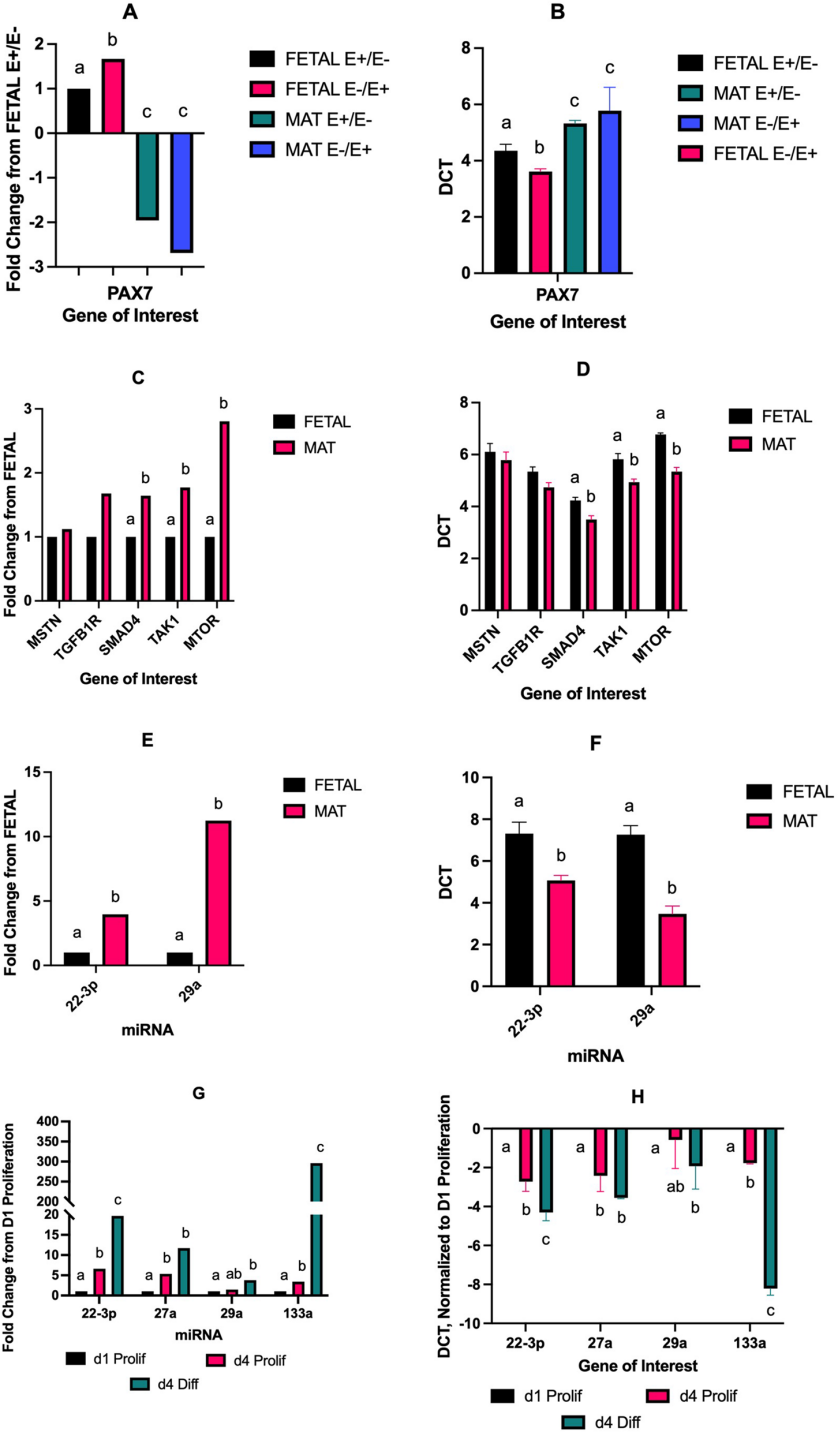
Longissimus muscle mRNA expression for PAX7 (**A**, **B**), other mRNA involved in muscle growth (**C**, **D**), and miRNA (**E**, **F**) by mycotoxin exposure (E + /E- vs. E-/E +) and developmental stage (FETAL, gestational d 133 vs. MAT, near maturity). miRNA expression during the proliferation and differentiation of ovine satellite cells (**G**, **H**). ^abc^Means with differing superscripts differ *P* < 0.05

miRNA sequencing was validated by qPCR (Fig. 7E, F). miR-22-3p and miR-29a were not affected (*P* > 0.05) by maternal fescue diet. miR-22-3p and 29a expression levels were increased (*P* < 0.001) for MAT samples compared to FETAL samples, in agreement with miRNA sequencing results.

Expression levels of genes known to be influential in muscle growth and development were examined by qPCR (Fig. 7). The satellite cell marker, PAX7, had a two-way interaction (*P* < 0.05) between maternal treatment and developmental age (Fig. 7A, B). Fetal E-/E + muscle had increased PAX7 expression when compared to E + /E- samples; whereas PAX7 expression did not differ by fescue treatment at the MAT time point. The genes SMAD4, TAK1, and MTOR were upregulated (*P* < 0.05) for MAT samples when compared to FETAL samples (Fig. 7C, D). The expression of MSTN and TGFB1R were not different (*P* > 0.10) by maternal treatment or developmental age (Fig. 7C, D).

### In vitro

Expression of miR-29a, 27a, 22-3p, and 133a in satellite cells during proliferation and differentiation is shown in Fig. 7G (fold-change) and 7H (delta C_T_). In general, miRNA expression increased (*P* < 0.05) as satellite cells proliferated and then differentiated to form myotubes. miR-27a expression increased (*P* < 0.05) from d 1 to d 4 of proliferation and remained constant during differentiation. miR-29a increased (*P* < 0.05) during differentiation as compared to d 1 of proliferation but miR-29a expression did not differ during proliferation. miR-22-3p and miR-133a increased (*P* < 0.05) during both proliferation and differentiation. miR-22-3p was upregulated during proliferation by fivefold and during differentiation by 20-fold. miR-133a was upregulated by 300-fold at d 4 of differentiation compared to d 1 of proliferation.

## Discussion

The *longissimus thoracis et luborum* muscle is the largest muscle in the lamb carcass [21, 22]. At d 133 of gestation the longissimus is 2.5% of empty body weight and at a mature market weight the longissimus is increased to 3.5% of the empty body weight [18, 19]. These studies also concluded that LATE gestion exposure to mycotoxins altered muscle growth in utero that caused long-term phenotypic changes during postnatal growth. From gestational d 70 to parturition, roughly 80% of fetal growth occurs making this a critical time for development [23].

Fescue seed treatment during LATE (E-/E +) gestation did not alter muscle fiber type in the longissimus but did result in smaller muscle fibers at market weight even though fetal muscle fiber size was unchanged. These results show that exposure to mycotoxins during the LATE gestation period altered postnatal longissimus muscle growth of Type I and II fibers. Similarly, others reported that cross-sectional area of Type I and Type II fibers in the fetal (gestational d 133) semitendinosus muscle was not altered by exposure to mycotoxins fed during MID (E + /E-) or LATE gestation [18]. Duckett et al. (2014) found reduced cross sectional area for slow (Type I) and fast twitch (Type II) muscle fibers in the longissimus muscle at birth from lambs born to ewes consuming E + during both MID and LATE gestation. In contrast, maternal nutrient restriction from d 35—85 of gestation causes reduced fiber area in the longissimus of lambs at 17 weeks of age [24]. Other researchers [25] also observed that maternal nutrient restriction during the last 6 weeks of gestation alters muscle fiber area for Type I and II fibers in male offspring at market slaughter.

The results from the current study indicate that longissimus muscle mass increases during postnatal growth are due to extensive muscle fiber hypertrophy. Cross-sectional Type I and Type II longissimus muscle fiber area increased from FETAL to MAT by 6.86-fold and 10.83-fold, respectively. Cross-sectional area of beef cattle from birth to 24 months of age showed a 9 to tenfold increase in fiber size across 4 different breeds [26]. Additionally, cross-sectional area of longissimus muscle fibers increases 100% during gestation in sheep from d 90 to 135 of gestation [27].

Type I fiber number as a percentage was increased for MAT lambs compared to FETAL lambs. At birth, Type I fibers represent 10–20% of the total fibers and differences seen at the FETAL developmental stage could be due to the prenatal sampling time [28]. The ratio of secondary (Type II):primary (Type I) fibers for lamb at birth is 12:1 and for pigs it is 14:1 [7]. Skeletal muscle fibers can transition between Type I and II metabolic states [29]. Scheffler et al. [30] reported that muscle fiber hypertrophy and greater oxidative metabolism can occur during postnatal growth in pigs. These findings are consistent with increased cross-sectional area and increased percentage of Type I fibers during development.

Maternal mycotoxin exposure did not alter miRNA expression in the longissimus muscle of male lambs. These results are in contrast to previous reports where maternal fescue treatment altered a small number of miRNA expressed in semitendinosus muscle tissue of fetal lambs [18]. Muscles used for locomotion, such as the semitendinosus, tend to have an increased percentage of Type II fibers compared to muscles used for posture such as the longissimus [28]. The difference in fiber types and fiber metabolisms between hindlimb muscles and trunk muscles may contribute to miRNA expression differences seen in the current study.

Developmental age did have an impact on the miRNA profile of the longissimus. Several miRNA identified in the current study have been previously identified in skeletal muscle. miR-133 is considered a muscle specific miRNA because it is preferentially expressed in skeletal muscle tissue [31]. miR-133 is reported to promote myoblast proliferation and fusion in addition to muscle growth [31]. miR-29a is involved with satellite cell activation and proliferation, and by extension muscle hypertrophy [32]. The conversion of oxidative fibers to glycolytic is facilitated by miR-22-3p [33]. miR-22-3p has been previously identified in ovine muscle tissue [18]. An overexpression of miR-410 has been shown to increase myoblast proliferation [34]. The current results show that dramatic changes occur in the miRNA transcriptome of the longissimus during the period of prenatal to postnatal muscle hypertrophy and these sequencing results were confirmed by qPCR.

KEGG pathway analysis showed enrichment of the PI3K-ATK pathway. The PI3K-AKT pathway has been shown to be directly involved with myoblast proliferation and muscle growth in chick embryos, and inhibition of the pathway results in reduced muscle mass [35]. The current study showed that the PI3K-AKT pathway had several enriched genes that could contribute to increases in muscle hypertrophy.

Additional assessment of mRNA known to be influential in muscle growth and hypertrophy or miRNA production and maturation were assessed using qPCR. PAX7 is a marker for satellite cells, muscle progenitor cells [36]. Increased expression of PAX7 is indicative of a larger satellite cell population in the muscle tissue, which is consistent with previous literature examining satellite cell populations during muscle development [37, 38]. Mycotoxin exposure during LATE gestation increased PAX7 expression at the FETAL developmental stage, however, muscle mass was not increased postnatally. PAX7 expression can be variable within the satellite cell population, and cells producing more PAX7 are less likely to activate and fuse with myofibers to increase hypertrophy [39]. The MSTN protein, a member of the TGFB super family, is a negative regulator of muscle growth and can bind with TGFBR1 protein [40]. Neither of these genes were altered by mycotoxin exposure or animal age indicating that they are consistently expressed during development and show some tolerance of environmental factors. The protein SMAD4 acts in the downstream signaling of MSTN to activate the transcription of target genes [40]. Additionally, TAK1 acts in the p38 MAPK signaling pathway, in response to MSTN, independently of SMAD signaling [41]. The MTOR signaling pathway has been shown to be involved in muscle growth and development as well as muscle related diseases through a variety of signaling ligands including MSTN [42]. While MSTN expression was not altered by developmental age, downstream signaling pathway proteins were altered by advancing animal development and muscle hypertrophy.

Ovine satellite cells were cultured to determine changes in miRNA expression during proliferation and differentiation to gain a better understanding of how they are involved in muscle growth. miRNA expression changes during the proliferation and differentiation phases of satellite cell development [43]. Postnatal muscle growth consists of hypertrophy directly influenced by satellite cell populations [39]. The majority of miRNA are upregulated during satellite cell differentiation [39]. miR-22-3p overexpression promotes myoblast differentiation and reduces proliferation [44]. miR-133a has been shown to indirectly target solute carrier family 2 member 4 (SLC2A4 or GLUT4) and increase cell proliferation and differentiation in chick skeletal muscle [45]. miR-27b increases satellite cell proliferation by targeting the negative regulator of muscle growth MSTN [46]. Mouse myoblast proliferation is inhibited by miR-29a [47]. miR-29a targets Protein Kinase B gamma and reduces the expression of mRNA and protein abundance. McFarlane et al. [8] showed that MSTN regulates satellite cell self-renewal via extracellular signal-regulated kinase 1/2 mitogen activated protein kinase pathway and the subsequent regulation of PAX7. In the current study, miRNA expression increased from day 1 of proliferation to day 4 for miR-27a, 22-3p, and 133a. Additionally, miR-29a, 22-3p, and 133a expression increased during satellite cell differentiation. These results indicate that these miRNAs are involved in satellite cell proliferation and differentiation and, by extension, muscle fiber hypertrophy.

## Conclusions

Mycotoxin exposure during LATE gestation had long-term impacts that resulted in lower body weight and longissimus muscle weight at MAT age. These differences in muscle mass were associated with reductions in the cross-sectional area or hypertrophy of the muscle fiber in lambs exposed to mycotoxins during LATE gestation. These results indicate that mycotoxin exposure during LATE gestation can alter postnatal muscle hypertrophy in sheep; however, these changes do not appear to be regulated by the miRNA profile in the longissimus muscle. Many miRNAs were differentially expressed during postnatal growth. In vitro satellite cell cultures showed that miR-29a, -27a, -22-3p, and -133a are elevated during proliferation and differentiation, indicating that these miRNAs are involved in postnatal skeletal muscle hypertrophy.

## Materials and methods

### Experimental design

All animal experimental procedures were reviewed and approved by the Clemson University Institutional Animal Care and Use Committee (AUP 2014–081). All methods are reported in accordance with ARRIVE guidelines for the reporting of animal experiments. Pregnant ewes were randomly assigned to mycotoxin treatments (E +  = 1.77 mg/head/d of ergovaline/ergovalinine or E- = 0 mg/head/d of ergovaline/ergovalinine) that were fed during mid- (gd35-85; E + /E-, control, MID) or late- (gd86-133 or parturition; E-/E + , LATE) gestation [19, 48]. In experiment one, pregnancies (*n* = 8/mycotoxin treatment; E + /E- and E-/E +) were terminated at gd133 to obtain fetal measurements and longissimus muscle samples (FETAL developmental stage). In experiment two, pregnant ewes (*n* = 27) were randomly assigned to a mycotoxin treatment [E + /E- (MID) or E-/E + (LATE)] and went to term (average of 145 d). Lambs remained with the dam until weaning at 75 d of age. Castrated male lambs (*n* = 10/mycotoxin treatment) were then individually fed a high concentrate diet until they reached 56 kg at an average of 236 d of age (MAT, maturity, developmental stage) and were harvested to obtain carcass measurements and longissimus muscle samples. A schematic diagram of the maternal treatment and developmental stage at sample collection is provided in Supplemental Table 2.

Longissimus muscle samples were collected from a subsample of lambs (*n* = 12 total; *n* = 3/treatment/developmental stage) that were representative of the mean body weight for each mycotoxin treatment at that developmental stage for miRNA sequencing and muscle fiber typing. Longissimus muscle samples were immediately frozen in liquid nitrogen and stored at -80 °C for subsequent ribonucleic acid (RNA) extraction. Longissimus muscle samples were also placed in a form and covered in optimal cutting temperature solution (Fisher Scientific, Waltham, MA), snap frozen in liquid nitrogen, and stored at -80 ºC for subsequent histology processing. Additional details on experimental design is available [18, 19, 48].

### Muscle fiber histology

Samples were collected and processed using methods previously established with modifications [18]. Muscle samples were cryosectioned at a thickness of 10 µm. Two sections per animal were used for analysis of fiber types. Cryosections of muscle samples were stained using primary antibodies to myosin heavy chain (MHC)-fast (My-32 mouse IgG1, Abcam Cat# ab51263, RRID:AB_2297993), MHC-slow (BA-F8 mouse IgG2b, Hybridoma Bank, DHSB Cat# BA-F8, RRIDAB_10572253), MHC-Type IIa (SC-71 mouse IgG1, Hybridoma Bank, DHSB Cat# SC-71, RRID:AB_2147165), and MHC-Type IIx (6H1 mouse IgM, Hybridoma Bank, DHSB Cat# 6H1, RRID:AB_1157897), and Alexa Fluor 546 goat anti-mouse IgG1 secondary antibody (Thermo Fisher Scientific Cat# A-21123, RRID:AB_2535765), Alexa Fluor 488 goat anti-mouse IgG2b secondary antibody (Thermo Fisher Scientific Cat# A-21141, RRID:AB_2535778), or Alexa Fluor 488 goat anti-mouse IgM secondary antibody (Thermo Fisher Scientific Cat# A-21042, RRID:AB_2535711), respectively. Sections were also counterstained with DAPI at 5 µg/mL to highlight nuclei (Invitrogen) and Alexa Fluor 633 wheat germ agglutinin at 10 µg/mL (Invitrogen) to outline muscle fibers [49]. Stained muscle sections were mounted in Prolong Gold and samples were imaged using a GE INCell Analyzer 2500HS widefield microscope system (Cytiva, Marlborough, MA) equipped with a Nikon 20X objective (N.A. = 0.75). At least ten unique sample regions were imaged per section using a BGOFR filter cube (DAPI, blue, Ex;Em 390/18;433/47), 488 nm (Alexa Fluor 488, green, Ex;Em 475/28;512/23), 532 nm (Alexa Fluor 546, orange, Ex;Em 542/27;588/47), and 635 nm (Alexa Fluor 633, magenta, Ex;Em 632/22;676/48). The number of Type I and II fibers were counted and cross-sectional area of myofibers was measured using ImageJ software (NIH, https://imagej.nih.gov/ij/docs/guide/146-1.html) by a single trained individual. Then, within the Type II fibers the proportion of Type IIa and IIx fibers was determined using ImageJ software by a single trained individual.

### miRNA sequencing

Total RNA was extracted from longissimus muscle using Trizol reagent (Invitrogen, Thermo Fisher Scientific, Waltham, MA) according to the manufacturer. RNA was treated with a DNA-free Kit (Ambion, Carlsbad, CA) according to the manufacturer to remove any genomic deoxyribonucleic acid (DNA) contamination. Total RNA was quantified using a Nanodrop 1000 spectrophotometer (Thermo Fisher). RNA integrity numbers (RIN) were generated using an Agilent 2100 Bioanalyzer (Agilent Technologies, Santa Clara, CA) and all RIN values were above 7.6. Total RNA was stored at -80ºC until being shipped on dry ice to Novogene (Durham, NC) for library preparation and sequencing.

A small RNA library was constructed from 3 μg total RNA per sample and index primers were added using NEBNext® Multiplex Small RNA Library Prep Set for Illumina® (NEB, USA.) according to the manufacturer’s recommendations. Library quality was evaluated with an Agilent Bioanalyzer 2100. Indexed samples samples were clustered using a cBot Cluster Generation System and a TruSeq SR Cluster Kit v3-cBot-HS (Illumina) according to the manufacturer’s instructions. The prepared libraries were then sequenced using an Illumina HiSeqX platform and 50 base pair single-end reads were generated.

A schematic diagram for miRNA sequencing analysis is presented in Supplemental Fig. 1. The total number of raw reads was 358,464,706 with a minimum of 23,135,302 reads per individual sample and all samples had a Q30 of > 98.39%. Reads containing > 10% N (0.020%), 5’ primer contaminants (0.047%), or did not contain the 3’ primer and the insert tag (3.759%) were excluded from the data. The 3’ primer sequence was trimmed and reads containing poly A/T/G/C (0.063%) were excluded using Novogene custom perl and python scripts. Reads smaller than 18 nucleotides or greater than 35 were removed from analysis (2.84%). The remaining reads (93.29%) were used for mapping.

Reads were mapped to the reference genome, Ovis aries (TEXEL) ensembl release 96, using Bowtie [50]. Known miRNA were identified with mirdeep2 [51] and srna-tools-cli [52] with miRBase 20.0 as a reference. RepeatMasker was used to remove reads from protein-coding genes, repeated sequences, rRNA, tRNA, snRNA, and snoRNA. Reads that went unmapped were analyzed using miREvo [53] and mirdeep2 based on characteristics of a hairpin structure: the secondary structure, Dicer cleavage site and minimum free energy of the small RNA to predict potentially new miRNAs. miRNA expression was normalized and estimated as transcripts per million [54]. Additional data analysis was provided by the Clemson University Genomics and Bioinformatics Facility. Specifically, target genes for miRNAs were predicted using miRanda [55]. The list of unique target messenger RNA (mRNA) was made from cleaning the list of predicted mRNA of duplicates. The most significant log_2_ fold change from miRNA differential expression that had a *P*_*adj*_ < 0.05 was retained for each unique mRNA and these values were used for enrichment analysis. Enrichment analyses of target mRNA predicted from differentially expressed miRNA, Gene Ontology (GO) and Kyoto Encyclopedia of Genes and Genomes (KEGG), were preformed using clusterProfiler [56].

### PCR

Complementary DNA (cDNA) for mRNA expression analysis was made with qScript cDNA SuperMix (Quanta Bio, Beverly, MA) according to the manufacturer and stored at -20ºC. PowerUp™ (ThermoFisher) SYBR™ green and a Quant Studio 3 Real-Time PCR System (ThermoFisher) were used for mRNA expression analysis according to the manufacturer. The polymerase chain reaction (PCR) program used was a standard program of 2 min at 50ºC, 2 min at 95ºC, and 40 cycles of 95ºC for 15 s, 60ºC for 15 s, and 72ºC for 1 min. Primers for PCR were designed to span exon boundaries using PrimerQuest™ tool (IDT; Coralville, IA; Table 2). Expression of mechanistic target of rapamycin (MTOR), transforming growth factor beta receptor 1 (TGFBR1), paired box 7 (PAX7), myostatin (MSTN), Smad4, and transforming growth factor beta activated kinase 1 (TAK1) were examined due to their involvement in muscle growth. Beta-actin (ACTB), glyceraldehyde 3-phosphate dehydrogenase (GAPDH), cyclophilin B protein (CYC), tubulin (TUB), and ribosomal protein S9 (RPS9) were examined as housekeeping genes using RefFinder [57]. ACTB and RPS9 were determined to be the most stable and used for geometric mean normalization.

**Table 2:** Primer sequences for qPCR analyses of mRNA involved in muscle growth and miRNA validation of sequencing data

Gene	Forward, 5’-3’	Reverse, 5’-3’	Efficiency
mRNA			
MTOR	GACCTTCTGCCTTCACAGATAC	CTCCTTCTTGACACAGCTTAGG	1.05
TGFBR1	ACCAGGACCACTGCAATAAA	AGTGCGGTTATGGCAGATATAG	1.05
Smad4	GGATGACCTCCGTCGTTTATG	TGATGCTCTGTCTTGGGTAATC	1.02
PAX7	GACCACAGGCTGAGAAAGAA	GACGCTATTTACAGGGCTAGAG	1.03
MSTN	AGTACGATGTCCAGAGAGATGA	TATCCACAGTTGGGCCTTTAC	1.02
TAK1	GACGCACATGACCAACAATAAG	AGCCCACATGATACGGAAAG	0.97
RPS9	GTGAGGTCTGGAGGGTCAAA	GGGCATTACCTTCGAACAGA	0.97
TUB	CGAGAGCTGTGACTGTCTGC	GGCATGACGCTAAAGGTGTT	1.01
CYC	GGTCATCGGTCTCTTTGGAA	TCCATCACACGATGGAA	1.01
ACTB	GGGCAGTGATCTCTTTCTGC	CTCTTCCAGCCTTCCTTCCT	1.03
GAPDH	GGGTCATCATCTCTGCACCT	GGTCATAAGTCCCTCCACGA	0.98
miRNA			
miR-22-3p	AAGCUGCCAGUUGAAGAACUG		1.00
miR-29a	UAGCACCAUCUGAAAUCGGUU		1.00

For miRNA gene expression, TaqMan miRNA reverse transcription kit (ThermoFisher) was used to convert miRNA to cDNA. TaqMan Small RNA Assay was used for miR-22-3p (assay no. 242214; catalog no. 444886; ThermoFisher) and miR-29a (assay no. 000412; catalog no. 4427975; ThermoFisher). miRNA sequences for *Ovis aries* were obtained from miRBase and used to find miRNA sequences that matched in the TaqMan assay database (ThermoFisher). For housekeeping gene, the U6 snRNA TaqMan Assay (assay no. 001973; catalog no. 4427975; ThermoFisher) was used as recommended by the manufacturer for normalization of miRNA gene expression. qPCR was conducted using the TaqMan PCR assay kit and a QuantStudio3 Real-Time PCR system according to manufacturer instructions. mRNA reactions were performed in duplicate and miRNA reactions were performed in triplicate.

### In vitro

To determine the role of the miRNA identified through sequencing, we conducted in vitro experiments to assess the expression of miR-29a, -22-3p, -133 and -27b during satellite cell proliferation and differentiation. Ovine satellite cells were isolated from the longissimus muscle of male lambs according to Li et al. [58] and Danoviz and Yablonka-Reuveni [59]. Cells were resuspended in a pre-plating media [Dulbecco’s modified eagle’s medium (DMEM; Gibco, Thermo Fisher) + 10% fetal bovine serum (FBS; Avantor, VWR, Radnor, PA) + 1% penicillin/streptomycin (Corning, VWR)] for 2 h in a T-75 flask to allow for removal of debris. After 2 h, cells were be transferred to a new T-75 flask for an additional 24 h to facilitate additional debris removal and increase cell population purity by selective adhesion [60]. Following pre-plating, satellite cells were plated on 0.1% gelatin coated T-25 plates for propagation, allowed to reach 80% confluence, and passaged twice. Additionally, an aliquot of cells was plated in a 0.1% gelatin coated 96 well plate at a density of 5,000 cells/well and allowed to plate for 24 h before being fixed and stained for PAX7 to assess population purity [61]. In brief, Cells were fixed and stained with a primary antibody for PAX7 (1:4, PAX7 mouse IgG1, Hybridoma Bank, DSHB Cat# PAX7, RRID:AB_528428) and goat anti-mouse IgG1 cross-adsorbed secondary antibody (1:1000, Thermo Fisher Scientific, Cat# A-10530, RRID:AB_2534035). Cells were also stained with Hoechst 33,342 (10 μg/mL, Thermo Fisher Scientific) to visualize the nucleus. Cells were imaged immediately using a Cytation1 (BioTek) with a 10X objective. At least 5 unique sample regions were imaged per well using a DAPI filer cube (DAPI, blue, EX;Em 377/50;447/60) and a Texas Red filter cube (Texas Red, red, Ex;Em 586/15;647/57). Satellite cell population purity was assessed as the number of cells expressing PAX7 protein divided by the total number of cells. Satellite cell population purity was 95%. Cells were pelleted following the propagation, counted with a hemocytometer, and frozen [DMEM (Gibco, ThermoFisher) + 20% FBS (Avantor, VWR) + 10% dimethylsulfoxide (Corning, VWR)] for later experiments.

Satellite cells were plated at 20,000 cells/well in 0.1% gelatin coated 24 well plates. Cultures were grown for 4 d in growth media [DMEM (Gibco, ThermoFisher), 20% FBS (Avantor, VWR), 1% penicillin/streptomycin (Corning, VWR), and 0.1% gentamicin (VWR)]. Cultures were differentiated for 4 d in differentiation media [DMEM low glucose (Gibco, ThermoFisher), 2% FBS (Avantor, VWR), 1% penicillin/streptomycin (Corning, VWR), and 0.1% gentamicin (VWR)]. Cells were collected for RNA extraction using Trizol at d 1 and 4 of proliferation and d 4 of differentiation. Experiments were run in duplicate.

### Statistical analysis

Histology data, and mRNA and miRNA qPCR results were analyzed as a 2 × 2 factorial using the GLM procedure of SAS with maternal fescue treatment (E + /E- or E-/E +), developmental stage (FETAL or MAT), and two-way interactions as fixed effects in the model. For histology data, slide replicate was included as a random effect. Significance was determined at *P* < 0.05. Differential expression analysis of miRNA expression was conducted using DESeq R package (1.8.3) and *P* values were adjusted with the Benjamini and Hochberg method [62]. Significance was determined at *P*_*adj*_ < 0.05. Lamb was the experimental unit for all the above analyses. Satellite cell culture (*n* = 2) miRNA expression was analyzed at day 4 of proliferation and differentiation and compared to day 1 of proliferation as the control. Replicate was the experimental unit for the satellite cell culture data. Significance was determined at *P* < 0.05.

## Supplementary Information

**Additional file 1: Supplemental Figure 1. **Schematic diagram of miRNA sequencing analysis pipeline.

**Additional file 2: Supplemental Table 1. **Differential expression of miRNA in longissimus muscle by developmental stage (FETAL = gd133; MAT = near maturity).

**Additional file 3: Supplemental Table 2. **Schematic diagram of the treatment structure and sample collection times. Maternal mycotoxin treatments (E+ = 1.77 mg/head/d of ergovaline/ergovalinine or E- = 0 mg/head/d of ergovaline/ergovalinine) that were fed during mid- (gestational d 35-85; E+/E-, control, MID) or late- (gestational d 86-133 or parturition; E-/E+, LATE) gestation. FETAL samples were collected on d 133 of gestation, MAT samples were collected near maturity (56 kg body weight, average 236 d of age.

## Data Availability

The datasets generated and analyzed during the current study are available in Gene Expression Omnibus GEO (GSE195597).

## Abbreviations

ACTB: Beta-actin
AKT: Protein Kinase B
BW: Body weight
cDNA: Complementary DNA
CYC: Cyclophilin B protein
DMEM: Dulbecco’s modified eagle’s medium
DNA: Deoxyribonucleic acid
E-: Endophyte-free tall fescue
E +: Endophyte-infected tall fescue
FBS: Fetal bovine serum
GAPDH: Glyceraldehyde 3-phosphate dehydrogenase
GLUT4: Glucose transporter type 4
GO: Gene ontology
KEGG: Kyoto Encyclopedia of Genes and Genomes
MHC: Myosin heavy chain
miRNA: Micro ribonucleic acid
mRNA: Messenger ribonucleic acid
MSTN: Myostatin
MTOR: Mechanistic target of rapamycin
PAX7: Paired Box 7
PBS: Phosphate Buffered Saline
PCR: Polymerase chain reaction
PI3K: Phosphoinositide 3 kinase
qPCR: Quantitative polymerase chain reaction
RIN: RNA integrity number
RNA: Ribonucleic acid
RPS9: Ribosomal protein S9
TAK1: Transforming growth factor beta activated kinase 1
TGFBR1: Transforming growth factor beta receptor 1
TUB: Tubulin
