# Supplementary material for: Impact of fetal exposure to mycotoxins on longissimus muscle fiber hypertrophy and miRNA profile
Source: BMC Genomics. 2022 Aug 16;23:595. doi: 10.1186/s12864-022-08794-0 (PMC9380335; doi:10.1186/s12864-022-08794-0)
Supplement: Supplementary file 1 — Additional file 1: Supplemental Figure 1. Schematic diagram of miRNA sequencing analysis pipeline. [file 12864_2022_8794_MOESM1_ESM.docx]

**Supplemental Figure 1.** Schematic diagram of miRNA sequencing analysis pipeline.

Raw Data

Quality control and data cleaning

Reference-based Mapping

sRNA classification and quantification

Known miRNA

Novel miRNA prediction

Differential expression analysis

Targeted gene prediction of differentially expressed miRNA

GO enrichment

KEGG enrichment
