# Supplementary material for: Impact of fetal exposure to mycotoxins on longissimus muscle fiber hypertrophy and miRNA profile
Source: BMC Genomics. 2022 Aug 16;23:595. doi: 10.1186/s12864-022-08794-0 (PMC9380335; doi:10.1186/s12864-022-08794-0)
Supplement: Supplementary file 2 — Additional file 2: Supplemental Table 1. Differential expression of miRNA in longissimus muscle by developmental stage (FETAL = gd133; MAT = near maturity). [file 12864_2022_8794_MOESM2_ESM.docx]

**Supplemental Table 1.** Differential expression of miRNA in longissimus muscle by developmental stage (FETAL = gd133; MAT = near maturity).

| **miRNA** | **FETAL** | **MAT** | **log_2_FoldChange** | ***P_adj_*** |
| --- | --- | --- | --- | --- |
| oar-miR-22-3p | 318.75 | 10513.29 | 4.94 | 5.15E-37 |
| novel_120 | 0.26 | 730.17 | 11.16 | 1.12E-36 |
| oar-miR-487b-3p | 1243.00 | 143.64 | -3.18 | 2.17E-36 |
| oar-miR-3958-3p | 11207.53 | 971.52 | -3.57 | 1.12E-35 |
| oar-miR-29a | 87.51 | 3463.21 | 5.22 | 3.93E-27 |
| oar-miR-410-3p | 1930.48 | 54.70 | -5.19 | 2.66E-23 |
| oar-miR-299-5p | 1535.81 | 75.77 | -4.39 | 1.25E-22 |
| oar-miR-382-5p | 4441.51 | 376.15 | -3.60 | 6.25E-20 |
| oar-miR-655-3p | 2798.64 | 281.79 | -3.37 | 1.25E-17 |
| oar-miR-3955-5p | 367.80 | 42.76 | -3.15 | 1.62E-17 |
| oar-miR-381-3p | 311636.63 | 15890.61 | -4.33 | 3.37E-16 |
| oar-miR-412-3p | 1676.67 | 173.82 | -3.33 | 5.38E-15 |
| oar-miR-26a | 70228.61 | 568536.00 | 2.94 | 9.10E-15 |
| oar-miR-323a-3p | 1061.17 | 139.62 | -2.94 | 4.01E-14 |
| oar-miR-29b | 2.36 | 145.02 | 5.89 | 4.24E-14 |
| oar-miR-133 | 45127.92 | 791320.52 | 4.02 | 2.44E-13 |
| oar-miR-329b-3p | 989.34 | 100.74 | -3.37 | 2.44E-13 |
| oar-miR-3956-5p | 1728.87 | 50.08 | -5.13 | 2.44E-13 |
| oar-miR-299-3p | 296.16 | 24.63 | -3.64 | 2.81E-13 |
| oar-miR-409-5p | 2923.34 | 412.15 | -2.91 | 4.28E-13 |
| oar-miR-26b | 5956.55 | 46475.16 | 2.88 | 5.05E-13 |
| oar-miR-493-5p | 13627.71 | 1641.88 | -3.06 | 5.05E-13 |
| oar-miR-543-3p | 44560.79 | 1245.40 | -5.20 | 4.78E-12 |
| oar-miR-495-3p | 22727.38 | 1384.91 | -4.07 | 8.01E-12 |
| oar-miR-30b | 642.26 | 12782.45 | 4.27 | 1.07E-11 |
| oar-miR-200c | 5.24 | 299.49 | 5.85 | 2.12E-11 |
| oar-miR-432 | 5721.91 | 486.34 | -3.61 | 5.02E-11 |
| oar-miR-191 | 3139.18 | 13722.59 | 2.06 | 5.92E-11 |
| novel_135 | 3.21 | 384.33 | 6.80 | 5.05E-10 |
| oar-miR-30c | 8262.43 | 48407.00 | 2.49 | 7.91E-10 |
| oar-miR-154b-5p | 1597.43 | 149.53 | -3.46 | 8.14E-10 |
| oar-miR-323c | 489.81 | 43.68 | -3.52 | 9.77E-10 |
| oar-let-7g | 58347.78 | 338093.97 | 2.45 | 1.52E-09 |
| oar-miR-30a-5p | 8982.74 | 46916.02 | 2.32 | 1.52E-09 |
| oar-miR-3956-3p | 170.41 | 3.09 | -5.93 | 2.08E-09 |
| oar-miR-194 | 62.11 | 1008.77 | 3.95 | 2.57E-09 |
| oar-miR-21 | 6009.73 | 55306.67 | 3.18 | 3.43E-09 |
| oar-miR-200b | 5.04 | 603.62 | 6.69 | 4.25E-09 |
| oar-miR-30a-3p | 921.29 | 3521.47 | 1.88 | 8.54E-09 |
| oar-miR-495-5p | 92.87 | 5.88 | -4.02 | 9.19E-09 |
| oar-miR-30d | 57061.00 | 293347.83 | 2.28 | 9.53E-09 |
| oar-miR-433-3p | 1453.73 | 94.45 | -4.00 | 9.72E-09 |
| oar-miR-409-3p | 7688.57 | 1096.49 | -2.86 | 2.52E-08 |
| oar-miR-493-3p | 13130.62 | 1201.42 | -3.45 | 3.16E-08 |
| oar-miR-370-3p | 12706.90 | 1022.69 | -3.68 | 2.13E-07 |
| oar-miR-374a | 49.23 | 355.61 | 2.83 | 2.89E-07 |
| oar-miR-200a | 6.01 | 122.97 | 4.28 | 1.50E-06 |
| oar-miR-150 | 205.57 | 1313.13 | 2.60 | 1.54E-06 |
| oar-miR-370-5p | 58.06 | 6.28 | -3.40 | 1.54E-06 |
| oar-miR-411a-3p | 1394.54 | 177.43 | -2.94 | 1.75E-06 |
| oar-miR-329b-5p | 51.21 | 1.45 | -4.93 | 3.10E-06 |
| oar-miR-10b | 57618.82 | 449137.91 | 3.01 | 4.87E-06 |
| oar-miR-3958-5p | 55.96 | 2.20 | -4.56 | 5.73E-06 |
| oar-miR-487b-5p | 67.15 | 4.36 | -4.23 | 6.29E-06 |
| oar-miR-23b | 2996.86 | 9377.12 | 1.56 | 1.01E-05 |
| novel_181 | 6.06 | 32.27 | 2.36 | 1.14E-05 |
| oar-miR-431 | 102.77 | 14.36 | -2.89 | 1.26E-05 |
| oar-miR-23a | 2400.07 | 7400.03 | 1.54 | 1.85E-05 |
| novel_331 | 2.11 | 25.96 | 3.53 | 2.38E-05 |
| oar-miR-106b | 45.61 | 189.36 | 2.02 | 2.64E-05 |
| oar-miR-380-3p | 4307.96 | 797.11 | -2.52 | 2.80E-05 |
| oar-miR-485-5p | 1437.73 | 199.18 | -2.91 | 4.86E-05 |
| oar-miR-3957-5p | 120.45 | 16.95 | -2.89 | 6.18E-05 |
| novel_129 | 68.92 | 11.60 | -2.56 | 7.20E-05 |
| oar-miR-17-5p | 84.19 | 322.75 | 1.90 | 1.31E-04 |
| oar-miR-103 | 1662.62 | 4491.67 | 1.34 | 1.83E-04 |
| novel_459 | 0.55 | 18.78 | 5.05 | 3.53E-04 |
| oar-miR-377-5p | 26.12 | 2.89 | -3.08 | 4.40E-04 |
| oar-miR-154a-3p | 212.76 | 71.13 | -1.69 | 4.49E-04 |
| novel_471 | 0.00 | 5.03 | 6.17 | 4.95E-04 |
| novel_360 | 1.32 | 16.74 | 3.67 | 5.86E-04 |
| novel_390 | 1.33 | 11.26 | 2.99 | 6.13E-04 |
| oar-miR-99a | 78159.46 | 206838.65 | 1.34 | 6.13E-04 |
| oar-miR-381-5p | 33.49 | 1.75 | -4.06 | 8.25E-04 |
| novel_338 | 0.00 | 9.06 | 6.93 | 8.81E-04 |
| oar-miR-412-5p | 62.97 | 5.96 | -3.38 | 9.38E-04 |
| oar-let-7f | 104819.92 | 344427.45 | 1.65 | 1.10E-03 |
| oar-miR-134-3p | 20.84 | 1.25 | -3.96 | 1.35E-03 |
| oar-miR-3955-3p | 21.96 | 0.00 | -5.33 | 1.53E-03 |
| oar-miR-655-5p | 26.15 | 2.22 | -3.64 | 1.58E-03 |
| oar-miR-127 | 144072.41 | 38661.38 | -2.00 | 1.81E-03 |
| oar-miR-758-3p | 90.36 | 20.17 | -2.20 | 1.89E-03 |
| oar-miR-143 | 61134.47 | 224333.95 | 1.89 | 2.67E-03 |
| oar-miR-369-5p | 257.59 | 46.14 | -2.50 | 3.12E-03 |
| novel_732 | 0.92 | 9.88 | 3.41 | 3.44E-03 |
| oar-miR-16b | 1256.77 | 3293.00 | 1.30 | 4.55E-03 |
| novel_454 | 0.10 | 4.31 | 5.47 | 4.83E-03 |
| oar-miR-541-5p | 116.12 | 12.90 | -3.14 | 4.83E-03 |
| oar-miR-10a | 6979.64 | 27102.88 | 1.98 | 5.03E-03 |
| oar-miR-376a-5p | 11.76 | 1.43 | -3.16 | 7.75E-03 |
| oar-miR-106a | 13.83 | 60.45 | 2.13 | 7.83E-03 |
| oar-miR-374b | 203.34 | 562.57 | 1.58 | 8.97E-03 |
| novel_815 | 0.00 | 8.66 | 6.73 | 1.17E-02 |
| oar-miR-369-3p | 982.19 | 322.40 | -1.65 | 1.27E-02 |
| oar-miR-494-5p | 15.43 | 2.62 | -3.00 | 1.49E-02 |
| oar-miR-668-5p | 6.12 | 0.00 | -3.61 | 1.51E-02 |
| oar-miR-379-3p | 434.10 | 153.48 | -1.47 | 1.82E-02 |
| novel_348 | 59.46 | 241.78 | 2.08 | 1.85E-02 |
| oar-miR-539-5p | 5.02 | 0.00 | -3.34 | 1.85E-02 |
| oar-miR-199a-3p | 9720.52 | 19383.78 | 0.91 | 1.96E-02 |
| oar-miR-218a | 386.75 | 1278.74 | 1.70 | 1.96E-02 |
| oar-miR-411b-5p | 152.44 | 53.39 | -1.58 | 1.96E-02 |
| oar-miR-323b | 25.95 | 6.45 | -2.09 | 1.98E-02 |
| novel_409 | 0.19 | 4.47 | 4.58 | 2.39E-02 |
| oar-miR-411a-5p | 7032.71 | 2139.36 | -1.70 | 2.46E-02 |
| oar-miR-1197-3p | 30.77 | 8.14 | -1.94 | 2.54E-02 |
| novel_591 | 0.51 | 5.65 | 3.52 | 2.83E-02 |
| oar-miR-134-5p | 48.25 | 19.17 | -1.41 | 2.98E-02 |
| oar-miR-485-3p | 284.11 | 83.31 | -1.93 | 3.04E-02 |
| oar-miR-362 | 452.88 | 972.99 | 1.02 | 3.06E-02 |
| oar-miR-380-5p | 41.22 | 9.94 | -2.13 | 3.47E-02 |
| novel_198 | 5.83 | 32.52 | 2.42 | 3.78E-02 |
| oar-miR-25 | 7599.98 | 15366.17 | 0.95 | 4.13E-02 |
| oar-miR-487a-5p | 39.38 | 9.46 | -2.06 | 4.38E-02 |
| oar-miR-136 | 638.46 | 167.34 | -1.94 | 4.38E-02 |
| novel_363 | 0.05 | 4.20 | 5.28 | 4.48E-02 |
| oar-miR-323a-5p | 5.71 | 0.00 | -3.50 | 4.66E-02 |
| novel_388 | 0.10 | 4.47 | 5.10 | 4.84E-02 |
| novel_290 | 0.00 | 2.39 | 5.32 | 4.84E-02 |
| novel_674 | 0.73 | 6.32 | 3.09 | 4.89E-02 |
