# Supplementary material for: Impact of fetal exposure to mycotoxins on longissimus muscle fiber hypertrophy and miRNA profile
Source: BMC Genomics. 2022 Aug 16;23:595. doi: 10.1186/s12864-022-08794-0 (PMC9380335; doi:10.1186/s12864-022-08794-0)
Supplement: Supplementary file 3 — Additional file 3: Supplemental Table 2. Schematic diagram of the treatment structure and sample collection times. Maternal mycotoxin treatments (E+ = 1.77 mg/head/d of ergovaline/ergovalinine or E- = 0 mg/head/d of ergovaline/ergovalinine) that were fed during mid- (gestational d 35-85; E+/E-, control, MID) or late- (gestational d 86-133 or parturition; E-/E+, LATE) gestation. FETAL samples were collected on d 133 of gestation, MAT samples were collected near maturity (56 kg body weight, average 236 d of age. [file 12864_2022_8794_MOESM3_ESM.docx]

**Supplemental Table 2.** Schematic diagram of the treatment structure and sample collection times. Maternal mycotoxin treatments (E+ = 1.77 mg/head/d of ergovaline/ergovalinine or E- = 0 mg/head/d of ergovaline/ergovalinine) that were fed during mid- (gestational d 35-85; E+/E-, control, MID) or late- (gestational d 86-133 or parturition; E-/E+, LATE) gestation. FETAL samples were collected on d 133 of gestation, MAT samples were collected near maturity (56 kg body weight, average 236 d of age.

|  | **Maternal Mycotoxin Treatment^1^** | |
| --- | --- | --- |
| **Developmental stage samples were collected** | **E+/E-** | **E-/E+** |
| **FETAL** gestational day 133 | Pregnant ewes (n = 8)   - Subsample used for analysis (n = 3 fetuses; 1 male fetus/ewe) | Pregnant ewes (n = 8)   - Subsample used for analysis (n = 3 fetuses; 1 male fetus/ewe) |
| **MAT** near maturity | Pregnant ewes (n = 13)   - Castrated males (n =10) fed to 59 kg body weight   - Subsample used for analysis (n = 3 lambs) | Pregnant ewes (n=14)   - Castrated males (n = 10) fed to 59 kg body weight   - Subsample used for analysis (n = 3 lambs) |

^1^E+ (endophyte-infected tall fescue seed containing ergovaline/ergovalinine) or E- (endophyte-free tall fescue seed without ergovaline/ergovalinine) was fed to ewes individually from gestational day 35 to 85 or gestational day 86 to 133, which is denoted by the ‘/’.
